# A Rare Case of *Alternaria citri* Keratitis Successfully Treated with Voriconazole

**DOI:** 10.3390/jof11110805

**Published:** 2025-11-13

**Authors:** Maura Bertazzolo, Giorgia Boaretto, Elena Zani, Massimo Busin, Deborah Cruciani, Silvia Crotti, Chiara Poletti, Roberta Vatri, Alessandra Caracciolo, Cristina Lapucci

**Affiliations:** 1 Department of Microbiology, Bianalisi S.p.A., 20841 Carate Brianza, MB, Italy; giorgia.boaretto@bianalisi.it (G.B.);; 2 Laboratory Bianalisi S.p.A., 47121 Forlì, FC, Italy; 3 Department of Ophthalmology, Ospedali Privati Forlì “Villa Igea”, 47122 Forlì, FC, Italy; 4 Experimental Zooprophylactic Institute of Marche “Togo Rosati”, 06126 Perugia, PG, Italy; d.cruciani@izsum.it (D.C.); s.crotti@izsum.it (S.C.)

**Keywords:** *Alternaria citri*, fungal keratitis, Voriconazole, ocular infection

## Abstract

The genus *Alternaria* comprises several species of dematiaceous hyphomycetes known to cause opportunistic infections in humans. Over the past two decades, fungal infections have emerged as a significant cause of morbidity and mortality, particularly among immunocompromised individuals. Such infections often occur following disruption of the skin or corneal epithelial barrier, especially in patients with pre-existing ocular conditions or compromised immune status. This case report describes a rare instance of fungal keratitis (FK) caused by *Alternaria citri* in a 71-year-old male who presented with an acute onset of eye infection. The patient showed a favorable response to treatment with voriconazole.

## 1. Introduction

The genus *Alternaria* includes several species of melanized hyphomycetes (pigmented filamentous fungi) that can cause opportunistic infections in humans. Ocular infection typically occurs following disruption of the skin or corneal epithelial barrier, particularly in patients with pre-existing ocular conditions, immunocompromised states, or transplant recipients [1,2,3]. Occupational exposure to soil and waste, surgical or non-surgical trauma, topical or systemic corticosteroid treatment, and diabetes mellitus are recognized as potential predisposing factors [4,5,6].

Fungal keratitis (FK) is an infectious disease of the cornea, characterized by ocular pain, conjunctival injection, stromal inflammatory infiltrates, and, frequently, corneal ulceration [7,8]. It may be caused by a variety of fungal pathogens with variable susceptibility to antifungal drugs, much like bacteria exhibit differing resistance to antibiotics. FK is the fourth leading cause of corneal blindness worldwide [9], with an estimated 1.5–2.0 million new cases annually, particularly in developing countries and agricultural regions with tropical and subtropical climates [5]. Up to three-quarters of affected individuals may suffer permanent vision loss.

FK can result in severe complications, including marked corneal inflammation, ulceration, scarring, and even perforation. A major challenge in its management is the accurate identification of the causative fungal agent [4,10]. Timely and accurate diagnosis through microscopy and culture is essential, as infections caused by filamentous fungi are sight-threatening and often clinically indistinguishable from bacterial keratitis [2]. Ophthalmologists and microbiologists should increasingly consider fungi as potential etiologic agents, especially given their growing role in infectious keratitis globally.

Voriconazole has demonstrated good ocular penetration and has been shown to be effective in treating fungal keratitis, including infections caused by *Alternaria* species. This case report describes fungal keratitis due to *Alternaria citri* in a 71-year-old male who developed an acute onset of eye infection and showed a favorable outcome following treatment with voriconazole. To the best of our knowledge, no previous human cases or clinical isolates attributed to *Alternaria citri* have been reported in the literature.

## 2. Case Report

A 71-year-old Italian man, currently retired and formerly employed as a craftsman specialized in woodworking, presented in June 2024 to the ophthalmology department of Ospedali Privati Forlì “Villa Igea” (Forlì) with a corneal abscess and significant corneal thinning in the left eye. He reported no contact with pets but maintained a small vegetable garden.

His ocular history included a mushroom penetrating keratoplasty in 2018 for herpetic keratitis in the same eye. In 2022, he underwent posterior lamellar endothelial keratoplasty due to endothelial decompensation, followed by lateral tarsorrhaphy for a persistent epithelial defect. The right eye had a history of central retinal vein occlusion.

At admission, due to the high risk of corneal perforation, the patient underwent a therapeutic penetrating keratoplasty à chaud.

Empirical antibiotic therapy was initiated with Ganciclovir gel (two times daily), brimonidine tartrate (three times daily), dorzolamide and timolol (three times daily), latanoprost (once daily), chloramphenicol plus betamethasone and tetracycline (every two hours), and bromfenac (two times daily).

Additionally, broad-spectrum fortified antibiotic eye drops (Vancomycin, Amikacin, and Ceftazidime) and fortified antifungal (Amphotericin B) were administered every two hours.

## 3. Materials and Methods

A corneal swab was promptly collected and analyzed for major microbial agents, including fungi, for which Sabouraud Gentamicin Chloramphenicol (SGC) was used.

**Figure 1:**
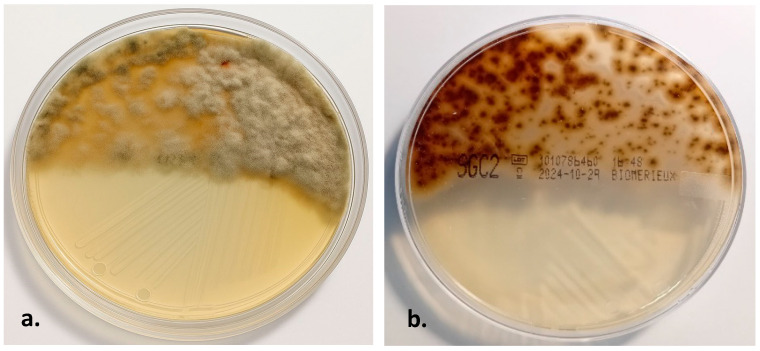
Growth plate SGC2 Biomerieux after 72 h. The macroscopic surface (**a**) colony appeared white to gray, turning dark olive. The macroscopic reverse (**b**) colony was blackish-brown.

Bacterial cultures were negative. After 48 h, only the Sabouraud Gentamicin Chloramphenicol (SGC2 Biomerieux) agar plate showed the growth of filamentous fungi (Figure 1).

For the microscopic evaluation of the colonies, the scotch technique with lactophenol blue dye was carried out. In order to determine the species, colonies grown on Sabouraud Dextrose Agar were subjected to PCR amplification and Sanger sequencing analysis.

**Figure 2:**
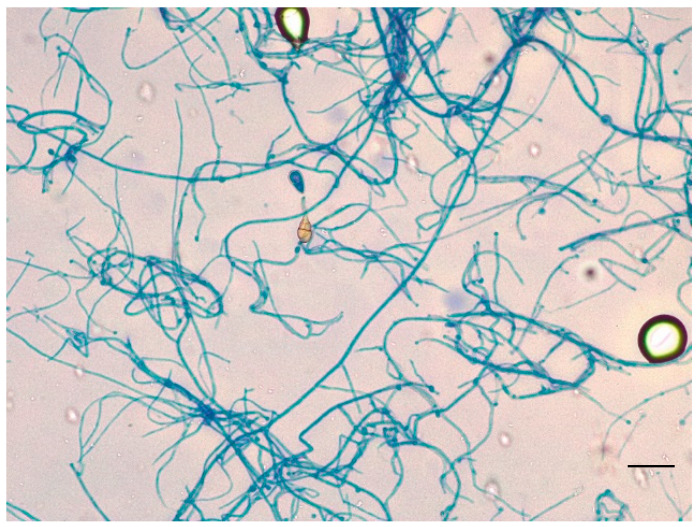
Growth plate after 48 h. Microscopic feature of the colonies with the technique of scotch and lactophenol blue dye (magnification 400×, bar 20 µm).

**Figure 3:**
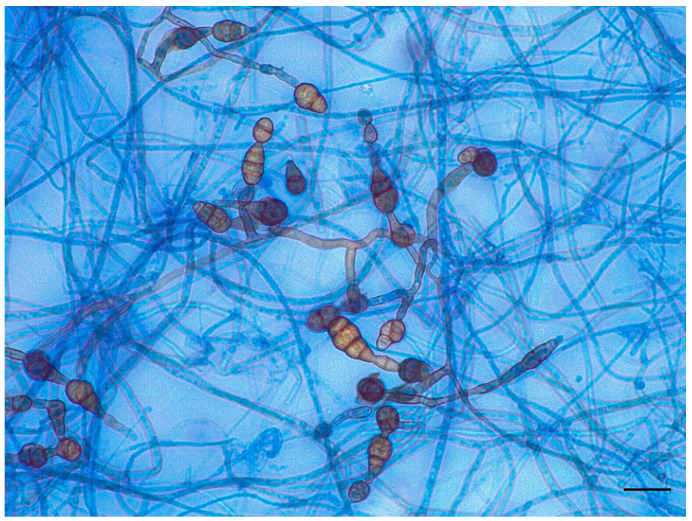
Growth plate after 4 days. The hyphae were pigmented and sectioned with chains of ovate conidia with transverse and longitudinal septa. Conidia were medium-brown, ovoid, broadly ellipsoid with transverse and longitudinal septa, and slightly or strongly constricted at the septa, in short to long, simple or branched chains (magnification 400×, bar 20 µm).

The identification of fungal growth was based on observation of macro- and microscopic features (Figure 2 and Figure 3).

In detail, one fungal colony was cut from the agar plate with a scalpel and transferred to a 1.7 mL tube. DNA was extracted by the QIAamp DNA mini kit (Qiagen, Hilden, Germany) following a modified Gram-positive protocol (Appendix D: Protocols for Bacteria, Isolation of genomic DNA from Gram-positive bacteria) preceded by an overnight step with lysozyme buffer. DNA was then subjected to PCR amplification of a portion of the internal transcribed spacer (ITS) region of ribosomal DNA using ITS4 (5′-TCCTCCGCTTATTGATATGC-3′) and ITS5 (5′-GGAAGTAAAAGTCGTAACAAGG-3′) primers [11]. DNA amplification was performed in a 50 µL volume consisting on 1× PCR Buffer, 1.5 mM MgCl_2_, 0.25 mM dNTPs, 0.5 µM of each primer, 1.25 U of Taq Hot Start (Promega, Madison, WI, USA), and 3 µL of DNA. The amplification thermal profile consisted of 95 °C for 10 min, followed by 35 cycles of 94 °C for 1 min, 53 °C for 1 min, 72 °C for 2 min, and a final extension at 72 °C for 7 min. PCR products were analyzed by gel electrophoresis on 2% agarose gel and visualized by Midori Green Advance DNA stain (NIPPON Genetics, Tokyo, Japan). PCR-positive reactions were purified using the QIAquick PCR Purification Kit (Qiagen). Sequencing was performed in both directions by BrilliantDyeTM Terminator v3.1 Cycle Sequencing Kit (NimaGen^®^, Nijmegen, The Netherlands). The reactions were separated through a 3500 Genetic Analyzer (Applied Biosystems^®^, Waltham, MA, USA), and the sequences were elaborated by BioEdit Sequence Alignment Editor software [12], version 7.2.5, to create a consensus sequence. Finally, it was aligned in the Westerdijk Fungal Biodiversity Institute database [13].

## 4. Results

After 48 h, the SGC plate showed growth of filamentous fungi. The macroscopic appearance of the colonies was analyzed in reverse and on the surface. Dematiaceous fungi, also known as “black molds”, are characterized by the presence of melanin pigments in the hyphal walls. Identification was based on the macroscopic observation of the colonies and evaluation of their microscopic features.

The colonies exhibited a velvety and soft texture. The macroscopic appearance of the colony was analyzed. The surface (Figure 1a) was white and gray that became dark olive. The reverse (Figure 1b) was blackish-brown.

Microscopically, after 48 h, with the technique of scotch and lactophenol blue dye, we observed very few ovate conida with transverse and vertical septa (Figure 2). After 4 days of incubation, we analyzed the colonies microscopically again. The hyphae were pigmented with mature muriform cells, sectioned with chains of ovate conidia with transverse and longitudinal septa. We could observe secondary conidiophores, apically or laterally, with a single conidiogenous locus (Figure 3) [14].

The initial identification was *Alternaria* spp., which was further specified as *Alternaria citri* by ITS sequencing, showing a 98.19% coverage, 100% sequence similarity, and zero probability of mismatch with *Alternaria citri* strain KY859404, based on the search conducted in the Westerdijk Fungal Biodiversity Institute database (UNITE community) [13]. Following the outcome of the investigations, voriconazole and natamycin were added to the patient’s treatment, with a progressive remission of the infection with no subsequent relapse up to a one-year follow-up. At 1 year, following complete suture removal, the graft remains clear with best-corrected Snellen visual acuity was 20/63.

## 5. Discussion

In recent years, phaeohyphomycoses have gained increasing clinical relevance as opportunistic fungal infections. *Alternaria* species are dematiaceous molds capable of causing oculomycosis, rhinosinusitis, onychomycosis, and cutaneous or subcutaneous infections, predominantly in immunocompromised hosts [15,16,17]. Nonetheless, infections in immunocompetent individuals have also been described, although invasive disease remains uncommon [1,6].

Fungal keratitis (FK) typically develops after disruption of the corneal epithelial barrier, particularly in individuals with predisposing ocular surface disease or chronic corticosteroid use. Occupational and environmental exposure, such as in farmers, gardeners, or woodworkers, represents an additional risk factor [2]. In the present case, the patient’s professional background as a woodworker and his ongoing gardening activities likely increased exposure to airborne fungal spores. Moreover, a history of multiple corneal surgeries and a persistent epithelial defect further compromised the corneal surface, facilitating fungal invasion.

Numerous cases of alternariosis have been attributed to *A. alternata*, *A. tenuissima*, and other related taxa. *A. alternata* is the most frequently reported species involved in human alternariosis and keratitis cases, followed by *A. tenuissima*. Species-level identification is clinically relevant because antifungal susceptibility and clinical outcomes vary among species [1].

Clinical response to therapy in *Alternaria* keratitis is heterogeneous. Early or superficial infections often respond to conventional topical antifungal agents such as natamycin or amphotericin B, whereas deep stromal or corticosteroid-associated infections may prove refractory [18]. Voriconazole, administered via topical, systemic, or intrastromal routes, has consistently demonstrated efficacy in such refractory cases. Recent reports document favorable outcomes with voriconazole, even after natamycin or amphotericin B failure, highlighting its importance as a therapeutic option for *Alternaria* keratitis [19].

Natamycin remains the only FDA-approved topical antifungal for filamentous keratitis; however, its limited corneal penetration restricts efficacy in deep stromal disease. Consequently, azoles such as voriconazole, which achieve superior corneal penetration, are frequently employed as first-line agents in recalcitrant infections [20]. In previously published series, early superficial infections responded well to natamycin, whereas deep stromal cases required adjunctive or alternative therapy with voriconazole (Table 1).

Traditionally, *Alternaria* spp. identification has relied on morphological features, including conidial structure, sporulation apparatus, and conidial chain formation [1,22,23]. Reliance on morphology alone, however, has led to considerable taxonomic uncertainty and frequent reclassification within *Alternaria* spp. and closely related genera (*Stemphylium*, *Embellisia*, *Nimbya*, and *Ulocladium*) [24]. Culture remains essential for accurate identification because *Alternaria* spp. readily grow on routine laboratory media, although clinically relevant isolates may lose their ability to sporulate in vitro.

Molecular approaches, including PCR-based assays and sequencing, have improved diagnostic accuracy by providing high sensitivity and specificity. Nevertheless, since pathogenic fungi can also colonize the normal ocular surface, molecular assays may occasionally yield false-positive results [21,25]. The Basic Local Alignment Search Tool (BLAST) (https://wi.knaw.nl/, accessed on 29 September 2024) enables comparison of unidentified fungal sequences, including non-sporulating isolates, against reference data in GenBank. However, approximately 14% of *Alternaria* spp. sequences in GenBank are misidentified [24]. Therefore, comparison against validated reference strains remains essential. Amplification and sequencing of the internal transcribed spacer (ITS) region using pan-fungal primers continue to represent reliable methods for *Alternaria* spp. identification.

Overall, *Alternaria* species exhibit good susceptibility to conventional antifungal agents. In selected cases, infection resolution has been achieved with corticosteroid tapering or surgical intervention alone. Historically, alternariosis has responded favorably to older antifungal agents [26,27]. *Alternaria* spp. isolates generally demonstrate in vitro susceptibility to posaconazole, terbinafine, and caspofungin, but resistance to micafungin has been reported, although data remain limited [2,28].

Recent prospective studies, however, have confirmed the efficacy and tolerability of voriconazole in severe keratomycosis, including *Alternaria* infections refractory to conventional therapy.

Voriconazole demonstrates excellent bioavailability and achieves therapeutic concentrations in both aqueous and vitreous humor after topical or oral administration, supporting its use as a first-line therapy for fungal keratitis [27,29,30]. Early initiation of antifungal therapy is critical to optimize visual outcomes. When medical treatment fails or infection progresses, prompt surgical management remains essential for infection control [30].

In the present case, the patient’s complex ocular history, including prior keratoplasties and a persistent epithelial defect, reduced the likelihood of medical therapy alone achieving resolution. Rapid progression of the corneal infiltrate and impending perforation necessitated urgent therapeutic penetrating keratoplasty. The combination of timely surgical intervention with targeted antifungal therapy ultimately resulted in complete clinical remission and absence of recurrence at one-year follow-up.

This case report is inherently limited by its single-patient design and the complexity of prior therapy. Before pathogen identification, the patient received broad-spectrum antibiotics and amphotericin B empirically. Although this empirical therapy may have contributed to transient stabilization, the marked improvement following targeted therapy strongly suggests that voriconazole and natamycin were decisive in achieving resolution. After microbiological confirmation, the combination regimen was maintained, leading to progressive healing and no relapse at one year.

This case adds to the growing evidence supporting voriconazole as an effective option for *Alternaria* keratitis, particularly in cases unresponsive to conventional agents.

## 6. Conclusions

*Alternaria* spp. are emerging etiologic agents of fungal keratitis in both immunocompromised and immunocompetent patients, often following ocular trauma or exposure to organic material. Rapid and accurate diagnosis using microscopy, culture, and molecular tools is essential for appropriate management, as clinical features often resemble bacterial infections. Voriconazole remains a valuable therapeutic option due to its ocular bioavailability and efficacy. Increased awareness among clinicians and microbiologists is critical to ensure timely diagnosis and improve visual outcomes in these potentially sight-threatening infections.

## Figures and Tables

**Table 1:** Comparative in vitro susceptibility patterns and reported clinical responses among *Alternaria* species implicated in keratitis. While *A. alternata* and *A. tenuissima* are the most frequently documented, this case represents the first report of *A. citri* keratitis.

Species	Typical Antifungal Susceptibility (In Vitro)	Common Clinical Therapy Reported	Observed Clinical Response/Outcomes	Key References
*A. alternata*	Generally susceptible to azoles (voriconazole, posaconazole, itraconazole) and terbinafine; variable sensitivity to amphotericin B; resistant to echinocandins (micafungin, caspofungin).	Topical natamycin ± oral/topical voriconazole; amphotericin B occasionally used.	Early or superficial infections respond to natamycin; deep stromal or refractory infections show better outcomes with voriconazole; keratoplasty occasionally required.	Pastor & Guarro (2008); Leite et al. (2023); Cuenca-Estrella et al. (2005–2006) [1,2,21]
*A. tenuissima*	Similar profile to *A. alternata*; sensitive to azoles; variable response to amphotericin B.	Natamycin or voriconazole (topical/systemic); keratoplasty in advanced disease.	Favorable outcomes with early voriconazole therapy; relapses uncommon after targeted treatment.	Hsiao et al.; Monno et al. (2015); Pastor & Guarro (2008). [1,17,18]
*A. citri* (present case)	Data extremely limited; presumed susceptible to voriconazole and natamycin based on genus-level patterns.	Voriconazole + natamycin after empirical antibiotics and amphotericin B.	Complete clinical remission, no relapse; causal attribution limited by prior empirical therapy.	Present study

## Data Availability

The original contributions presented in this study are included in the article. Further inquiries can be directed to the corresponding author.
